# Emissions of Nitrous Oxide and Nitric Oxide from Soils of Native and Exotic Ecosystems of the Amazon and Cerrado Regions of Brazil

**DOI:** 10.1100/tsw.2001.261

**Published:** 2001-11-21

**Authors:** Eric A. Davidson, Mercedes M.C. Bustamante, Alexandre de Siqueira Pinto

**Affiliations:** The Woods Hole Research Center, Woods Hole, MA 02543, USA

**Keywords:** deforestation, nitrogen, N_2_O, NO, pastures, savannas, tropical ecology

## Abstract

This paper reviews reports of nitrous oxide (N2O) and nitric oxide (NO) emissions from soils of the Amazon and Cerrado regions of Brazil. N2O is a stable greenhouse gas in the troposphere and participates in ozone-destroying reactions in the stratosphere, whereas NO participates in tropospheric photochemical reactions that produce ozone. Tropical forests and savannas are important sources of atmospheric N2O and NO, but rapid land use change could be affecting these soil emissions of N oxide gases. The five published estimates for annual emissions of N2O from soils of mature Amazonian forests are remarkably consistent, ranging from 1.4 to 2.4 kg N ha–1 year–1, with a mean of 2.0 kg N ha–1 year–1. Estimates of annual emissions of NO from Amazonian forests are also remarkably similar, ranging from 1.4 to 1.7 kg N ha–1 year–1, with a mean of 1.5 kg N ha–1 year–1. Although a doubling or tripling of N2O has been observed in some young (<2 years) cattle pastures relative to mature forests, most Amazonian pastures have lower emissions than the forests that they replace, indicating that forest-topasture conversion has, on balance, probably reduced regional emissions slightly (<10%). Secondary forests also have lower soil emissions than mature forests. The same patterns apply for NO emissions in Amazonia. At the only site in Cerrado where vegetation measurements have been made N2O emissions were below detection limits and NO emissions were modest (~0.4 kg N ha–1 year–1). Emissions of NO doubled after fire and increased by a factor of ten after wetting dry soil, but these pulses lasted only a few hours to days. As in Amazonian pastures, NO emissions appear to decline with pasture age. Detectable emissions of N2O have been measured in soybean and corn fields in the Cerrado region, but they are modest relative to fluxes measured in more humid tropical agricultural regions. No measurements of NO from agricultural soils in the Cerrado region have been made, but we speculate that they could be more important than N2O emissions in this relatively dry climate. While a consistent pattern is emerging from these studies in the Amazon region, far too few data exist for the Cerrado region to assess the impact of land use changes on N oxide emissions.

## INTRODUCTION

Nitrogen cycling in terrestrial ecosystems is affected by numerous factors, including geology, climate, historical land use, and current management practices. In recent decades, land use change has been occurring rapidly in the Amazon and Cerrado (savanna) regions of Brazil[1,2]. The magnitude of land use change in the

Amazon and Cerrado regions is sufficiently large that it could affect regional and global budgets of nitrous oxide ($N_2O$) and nitric oxide (NO) emissions from soils. $N_2O$ is a stable greenhouse gas in the troposphere[3], but participates in photochemical reactions in the upper atmosphere that destroy stratospheric ozone[4]. In contrast, NO participates in photochemical reactions in the troposphere that produce ozone, which is a greenhouse gas[5] and a pollutant that affects crop yield and human health[6].

Amazonian forests have been recognized as important sources of atmospheric $N_2O$ [7,8,9,10]. Significant emissions of NO have also been reported from both Cerrado soils[11] and Amazonian soils[10,12,13]. In the case of NO, however, deposition of nitrogen oxides ($NO_x$) onto forest canopy surfaces significantly reduces the amount emitted above the forest canopy[14]. In general, emissions of the more reduced form of nitrogen oxide, $N_2O$, are relatively more important in the Amazon region where the wet season is longer, whereas emissions of the more oxidized form, NO, are relatively more important in the Cerrado region where the wet season is shorter[10,15]. Although other factors can also be important at times, this broad-brush generalization regarding climate is fairly robust because $N_2O$ production from denitrification is usually favored in wet soils and NO production from nitrification usually dominates in drier soils[15,16,17].

Following a site disturbance such as deforestation, N availability in the soil often temporarily increases, causing significant increases in emissions of NO and $N_2O$[8,9,18]. However, reports of the length of this period of elevated emissions range from 10 years in lowland forests of La Selva, Costa Rica[18] to less than 6 months in the eastern Amazon region of Pará[10]. Less is known about the effects of land use change on N oxide emissions from Cerrado soils. Only recently have seasonal variations in emissions been adequately assessed to make reasonably reliable estimates of annual emissions for one site[19]. Frequent fires, both natural and anthropogenic, can prevent accumulation of ecosystem reserves of readily cycling N in the Cerrado. The most common conversion of Cerrado is to soybeans and cattle pasture.

The objective of this paper is to review the literature, particularly that which has become available within the last 2 years, regarding soil emissions of NO and $N_2O$ in the Amazon and Cerrado regions. The effects of land use change on emissions of N oxides from soils of these two biomes are examined.

## NITROUS OXIDE FROM AMAZONIAN FORESTS

The five published estimates for annual emissions of $N_2O$ from Amazonian forest soils are remarkably consistent, ranging from 1.4 to 2.4 kg N ha$^{-1}$ year$^{-1}$, with a mean of 2.0 kg N ha$^{-1}$ year$^{-1}$ (Table 1). Given the notoriously spatially heterogeneous nature of $N_2O$ emissions from soils[20] and the diversity of soil types in Amazonia[21], this relatively narrow range is somewhat surprising. These authors reported significant within-site spatial heterogeneity and seasonal variation in fluxes, as has been commonly observed, but aggregation of data to annual estimates on a per-hectare basis yielded similar estimates for soils from a broad range of geographic regions of the Amazon.

Although Amazonian soils are diverse, a common feature appears to be a relative abundance of N cycling in Amazonian forest ecosystems[22]. An exception is very sandy soils, such as some of the soils studied near Manaus by Matson et al.[23]. The $N_2O$ fluxes that they measured during a 6-week period near

**TABLE 1**
**Emissions of $N_2O$ and NO from Mature Forest Soils in the Brazilian Amazon Region**

| Locations | $N_2O$ Emissions (kg N ha$^{-1}$ year$^{-1}$) | NO Emissions (kg N ha$^{-1}$ year$^{-1}$) | Reference |
|---|---|---|---|
| Manaus, Amazonas | 1.4 | | 55 |
| | 1.9 | | 8 |
| | | 1.4[a] | 12, 13 |
| Paragominas, Pará | 2.4 | 1.5 | 10 |
| Tapajos, Pará | 2.3[b] | 1.7[b] | 27 |
| Nova Vida, Rondônia | 1.9 | 1.4[c] | 9, 54 |

[a] Annual extrapolation made by Davidson and Kingerlee[31] from data published in Bakwin et al. [12] and Kaplan et al.[13].
[b] Annual extrapolation made here based on seasonal trends presented by Nepstad et al.[27]. The extrapolation follows the procedure of Verchot et al.[10], in which dry season and wet season means were calculated from all sample dates within the respective season and then each mean was extrapolated to half of a year.
[c] Annual extrapolation made here based on means of one set of dry season measurements and two sets of wet season measurements reported by Garcia-Montiel et al.[54], and assuming 120 days of dry season and 245 days of wet season.

Manaus were about five times lower at a sandy site than those of a nearby site with finer textured soils reported by Luizão et al.[8], shown in Table 1. Hence, annual emissions of $N_2O$ from very sandy soils in the Amazon Basin, such as Psamments and Spodosols, are likely to be lower than those shown in Table 1, but no year-round studies have yet been conducted.

As reviewed by Verchot et al.[10], the annual emissions from Amazonian soils reported in Table 1 are in the middle of the range reported for tropical forests (Table 2), with higher values reported for a more fertile soil in Costa Rica[24] and lower values for montane tropical forests. Lowland humid old-growth forests in Puerto Rico had $N_2O$ emissions ranging from 0.6 to 1.7 kg N $ha^{-1}$ $year^{-1}$[25].

Assuming that a mean of 2.0 kg N $ha^{-1}$ $year^{-1}$ is applicable for all 5.4 $km^2$ of forest in the Amazon Basin, the annual emissions from the soils are about 1.1 Tg N $year^{-1}$ (1 Tg = $10^{12}$ g N). This extrapolation must be made with caution, because we are unaware of measurements made in Amazonian regions outside of Brazil. A modeling approach yielded similar estimates of 1.3 Tg N $year^{-1}$ for the entire Basin, but with a much larger range of fluxes (<1 to 4 kg N $ha^{-1}$ $year^{-1}$)[9] than indicated by measurements reported in Table 1. The model simulations also show interannual variation, with higher emissions during wetter La Niña years (1.4 Tg N $year^{-1}$; [9]) and lower emissions during drier El Niño years (1.2 N $year^{-1}$; [26]). This interannual variation is likely to be larger at sites that are strongly affected by El Niño/La Niña variation, such as the Tapajos National Forest, near Santarém, Pará, where the unusually wet La Niña year of 2000 resulted in wet season $N_2O$ emissons that were about twice those of 1999[27].

## NITROUS OXIDE FROM CERRADO

Soil emissions of $N_2O$ from two types of Cerrado vegetation (*sensu stricto* and *campo sujo*) at the IGBE reserve research site near Brasília were below detection limits of the chamber and gas chromatrography systems[10,19]. Hence, the annual emissions were <0.4 kg N $ha^{-1}$ $year^{-1}$ and were probably near zero. This research site at the IGBE reserve near Brasília has soils that are common in the Cerrado region (Latossolos Vermelho-Escuro e Vermelho-Amarelo in the Brazilian system and Acrustox in the USDA taxonomy system), but Cerrado soils are diverse and it is possible that detectable emissions could be found in other geographical regions of the Cerrado. However, low $N_2O$ emissions are commonly observed in well-drained savannas[28,29]. Both nitrifying and denitrifying bacteria produce $N_2O$, but the largest emissions occur from denitrification under anaerobic conditions[30], which are uncommon in well-drained soils in relatively dry climates. Low N availability in Cerrado soils, presumably due largely to N losses from frequent fires, also contributes to low soil emissions of N oxide gases[19].

## NITRIC OXIDE FROM AMAZONIAN FORESTS

Estimates of annual emissions of NO from Amazonian forests are also remarkably similar, ranging from 1.4 to 1.7 kg N $ha^{-1}$ $year^{-1}$, with a mean of 1.5 kg N $ha^{-1}$ $year^{-1}$ (Table 1). As expected, NO emissions are lower than $N_2O$ emissions from most Amazonian soils, especially during the wet season, because of the moist conditions that favor reduction of NO to $N_2O$ before either gas escapes from the soil surface[15,17]. These estimates of annual emissions of NO are toward the high end of the range reported for all tropical forests[31], and one slightly exceeds the range (Table 2).

## NITRIC OXIDE FROM CERRADO

A far more difficult problem is estimating NO emissions from savanna soils. Davidson and Kingerlee[31] point out in their review of global soil emission that savanna ecosystems represent the largest and most variable soil sources of NO. Large fluxes are often observed immediately following precipitation events that end long dry periods and after burning, both of which are common in savanna regions. The pulse events can contribute significantly to annual fluxes, but quantifying their importance is a difficult measurement problem.

Pinto et al.[19] have recently demonstrated that wetting of dry soil in the Cerrado near Brasília causes an increase in emissions of a factor of ten or more, but that the pulse is short lived. Within 24 h, the fluxes had dropped to being only two to four times larger than in the unwetted soils, and after 3 days there was no longer a difference among treatments. Similarly, fire caused a doubling of NO emissions, but the effect disappeared within 5 days after the fire. Hence, for this site, these pulses are unlikely to add more than 10 to 20% to the estimate of annual emissions. Based on average measured emissions not related to such pulses (0.5 ng N $cm^{-2}$ $h^{-1}$), the annual NO emissions were 0.4 kg N $ha^{-1}$ $year^{-1}$ (the detection limit for NO is typically lower than for $N_2O$,

**TABLE 2**
**Ranges of Soil Emissions by Biome and Region**

| | $N_2O$ (kg N $ha^{-1}$ $year^{-1}$) | NO (kg N $ha^{-1}$ $year^{-1}$) | Reference |
|---|---|---|---|
| Moist tropical forests globally | 0.3 – 6.7 | 0.7 – 1.4 | 10, 31 |
| Amazon Basin (this synthesis) | 1.4 – 2.4 | 1.4 – 1.7 | |
| Savannas globally | No review | 0.1 – 10.0 | 31 |
| Brazilian Cerrado | Below detection | 0.4 | 19 |

and so this annual estimate is significantly different from zero). This is about five times lower than the estimate of Poth et al.[11] for some of the same research sites at the IGBE Cerrado reserve based on less frequent sampling during the dry season and based on more generous extrapolations of the effects of fire and wetting events.

Similar "background" fluxes have been made for savannas of the world[28,29,32,33,34,35,36,37]. Considerably higher emissions have been reported for nutrient-rich savannas of part of Venezuela[38,39]. The largest differences in annual estimates and probably the greatest source of uncertainty, however, is the way that various authors have "scaled up" the transient effects of rainfall events and fire. Davidson and Kingerlee[31] accepted the reported annual estimates and calculated a mean of 3.1 kg N $ha^{-1}$ $year^{-1}$ with a range of 0.1 to 10.0 kg N $ha^{-1}$ $year^{-1}$ for tropical savannas of the world (Table 2). The Brazilian Cerrado covers two of the $24 \times 10^8$ ha of tropical savanna. If the site studied by Pinto et al.[19] is characteristic of the Brazilian Cerrado (the *stricto sensu* and *campo sujo* vegetation types that they studied cover 65% of the Cerrado biome) and perhaps also representative of large areas of the world's "nutrient-poor" savannas, and if their assessment of the minimal impact of transient effects of fires and precipitation are widely applicable, then the large estimate of NO emission from savannas made by Davidson and Kingerlee[31] may need to be adjusted downward. Many of these savannas are in rural areas distant from industrial and transportation inputs of $NO_x$, so the soil source of NO may be critical to the surrounding local and regional photochemistry of the atmosphere[40]. Hence, soil emissions of NO from tropical savannas such as the Brazilian Cerrado remain a large and important uncertainty.

## EFFECTS OF LAND USE CHANGE ON NITROUS OXIDE EMISSIONS

The classic deforestation experiments at Hubbard Brook[41], New Hampshire, U.S., demonstrated that eliminating the plant sink for nutrients causes a pulse of nutrient availability in soils and streams, including N. Soil emissions of $N_2O$ and NO are often positively correlated with N availability[15,17]. The magnitude and duration of pulses of N availability and N oxide emissions following site disturbance varies widely in tropical forests (Table 3), and probably depends upon the prior site fertility (i.e., the stocks of potentially mineralizable N) and the rate of vegetation regrowth after the disturbance event. Enhanced soil emissions of $N_2O$ and NO were measured up to 10 years after forest-to-pasture conversion near La Selva, Costa Rica[18], apparently due to the relatively N-rich soils that mineralized N in excess of pasture plant demands for a decade. Similar chronosequence studies in Rondônia showed that the period of higher emissions (3.1 to 5.1 kg N $ha^{-1}$ $year^{-1}$) was only 2 years[9]. In contrast, no elevated emissions were detected during either wet or dry seasons in pastures as young as 6 months following forest clearing and burning in eastern Pará[10].

A comparison of N availability assays confirmed that the Costa Rican site is more fertile than the eastern Brazilian site in Pará[15]. A direct comparison has not been made between the Paragominas (Pará) soils and Nova Vida soils (Rondônia), but published values of N availability assays appear similar. The Paragominas soils are finer textured and water content does not vary greatly between forest and pasture soils, whereas the Nova Vida soils are more coarsely textured and have higher water contents in pastures than in forests. We do not know if the different responses to deforestation in Rondônia and Pará sites were due to one of these edaphic factors, to management practices, or to a combination of the two. A goal of good pasture management throughout the region is quick grass establishment following clearing and burning. The exotic grasses commonly used in Amazonia appear to be good sinks for soil N, thus immobilizing the N released from the disturbance effect within months to a couple of years.

Old cattle pastures consistently produce less $N_2O$ than the native forests that they replaced[9,10,18,25], but the definition of "old" and the degree of reduction in emissions may vary regionally (Table 3). Melillo et al.[9] reported average emissions of 1.4 kg N $ha^{-1}$ $year^{-1}$ in pastures 4 to 41 years old, whereas Verchot et al.[10] reported a mean of 1.5 kg N $ha^{-1}$ $year^{-1}$ for a chronosequence of pastures 0 to 13 years old on one ranch and 0.2 kg N $ha^{-1}$ $year^{-1}$ for two pastures more than 25 years old on another ranch. Both studies included several sampling dates in both wet and dry seasons. Based on the assumption of higher emissions during the first 2 years of pasture growth and lower emissions thereafter, Melillo et al.[9] calculated that Basin-wide emissions of $N_2O$ have decreased about 0.02 to 0.05 Tg N $year^{-1}$ due to forest-to-pasture conversion. Using somewhat lower estimates of emissions from pastures, Verchot et al.[10] estimates a decrease of 0.02 to 0.08 Tg N $year^{-1}$. It is probably safe to say

**TABLE 3**
**Effects of Tropical Forest-Pasture Conversion on Soil Emissions of Nitrous Oxide**

| | (kg $N_2O$-N $ha^{-1}$ $year^{-1}$) | | | | |
|---|---|---|---|---|---|
| | Mature Forest | Young Pasture | Old Pasture | Secondary Forest | Reference |
| Amazonas, Brazil | 1.9 | 6 (3 years fertilized) | No data | No data | 8 |
| La Selva, Costa Rica | 6 | 35 – 50 (2 – 10 years) | 2 – 3 (18 – 25 years) | 4 | 18, 24 |
| Pará, Brazil | 2.4 | 1.5 (0 – 13 years) | 0.2 (25 years) | 0.9 | 10 |
| Rondônia, Brazil | 2 | 3 – 5 (1 – 3 years) | 1 – 2 (4 – 41 years) | No data | 9 |

that the Basin-wide effect of historical and continuing land use change is a reduction of emissions of <10% and probably not an increase in emissions.

These results contrast with earlier estimates that tropical deforestation has increased global emissions of $N_2O$ by 0.4 Tg N year$^{-1}$[42,43,44]. These estimates were based mostly on the results of Keller et al.[18] from Costa Rica and the study by Luizão et al.[8] of a pasture near Manaus that had been fertilized with N (Table 3). Fertilization with N is uncommon in Amazonian pastures, and more recent studies have clearly demonstrated that, in the long term, most Amazonian pastures emit less, not more, $N_2O$ than the forests that they replace. Given that the Amazon Basin is by far the largest of tropical forest regions, it is likely that the effect of tropical deforestation on the global $N_2O$ is very close to zero.

As deforestation proceeds and as degraded pastures are abandoned in the Amazon Basin, the area occupied by secondary forest is likely to increase. A 19-year-old secondary forest in eastern Pará had $N_2O$ emissions of about 40% those of the mature forest, but higher than adjacent old pastures, indicating only partial recuperation of N cycling processes[10]. In the Bragantina region of Pará, a secondary forest chronosequence on land previously used for slash-and-burn agriculture shows a trend of increasing emissions with secondary forest age, but even the 70-year-old forests had not yet achieved emission rates equal to 50% of the mature forest's emissions (Fig. 1). Hence, the reduction of $N_2O$ emissions caused by deforestation extends beyond the "old pasture" phase and can persist well into secondary forest succession phases[10,24,25] (Table 3). The regrowing forest is a sink for nutrients, including N, and we would expect N to become a relatively abundant nutrient only after biomass stocks of N stop aggrading appreciably. Atmospheric deposition inputs of N in the Amazon region are about 2 to 4 kg N ha$^{-1}$ year$^{-1}$[45,56], whereas aboveground biomass accumulation rates are 5 to 26 kg N ha$^{-1}$ year$^{-1}$[46,56], depending on the rate of regrowth. The abundance of leguminous species and presumably their inputs from biological N fixation are highly variable in tropical forests[25,47]. The rate at which $N_2O$ emissions recover during secondary succession may depend on such inputs[25], but this is poorly understood, especially for Amazonia.

The limited information currently available for the Cerrado indicates that conversion of native ecosystems to agricultural uses has increased $N_2O$ emissions modestly (Table 4). Modest increases above undetectable fluxes from Cerrado vegetation have been observed in cattle pastures. A 5-year-old cattle pasture had $N_2O$ emissions of 0.3 ng N cm$^{-2}$ h$^{-1}$ and 0.9 ng N cm$^{-2}$ h$^{-1}$ during the dry and wet seasons, respectively[48]. A 10-year-old pasture had $N_2O$ emissions of 0.2 ng N cm$^{-2}$ h$^{-1}$[49] during the wet season, and a 20-year-old pasture had emissions below detection limits at all times (Verella et al., unpublished data; Table 4). Although these three independent studies were not designed as a pasture chronosequence, and there are differences in grass species among them, the trend is strikingly similar to that observed in Amazonian pastures, where $N_2O$ emissions decrease with pasture age (Table 3). Planting of legumes is being encouraged by the government for recuperation of old degraded pastures, and N fertilization to recuperate pastures is also somewhat common, but no data are currently available on the effects of these practices on N oxide emissions.

Over 4 million hectares of Cerrado have been converted to soybean production (www.cnpso.embrapa.br), mostly using cultivars with high N fixation capacity[50]. Nobre[49] showed that $N_2O$ emissions increased during the first 100 days of soybean establishment, reaching 0.5 ng N cm$^{-2}$ h$^{-1}$, presumably as the result of increasing N fixation by the crop. In contrast, $N_2O$ emissions in cornfields were highest (1.5 ng N cm$^{-2}$ h$^{-1}$) shortly after fertilization at the beginning of the crop cycle and declined to 0.2 ng N cm$^{-2}$ h$^{-1}$ after 100 days[49] (Table 4). In another study of soybean fields in a soybean-corn crop rotation, emissions ranged from 0.8 to 2.5 ng N cm$^{-2}$ h$^{-1}$[48].

These increases are modest compared to other published accounts of elevated emissions from tropical agricultural

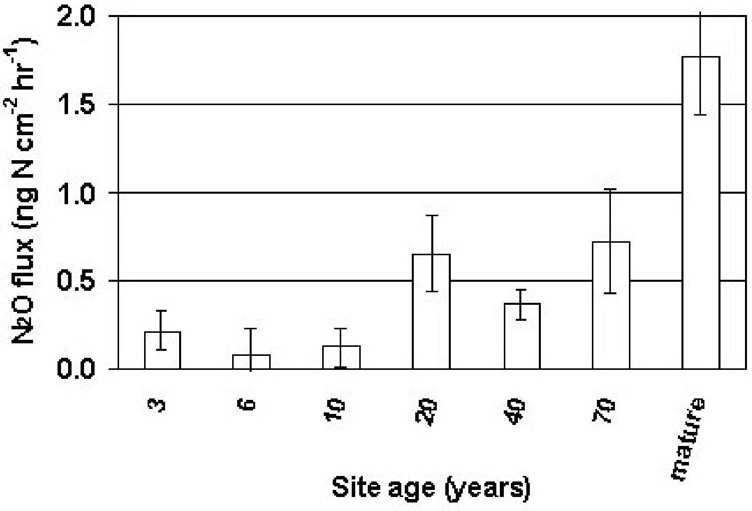

**FIGURE 1.** Soil emissions of $N_2O$ in a secondary forest chronosequence at São Francisco do Pará, Brazil. Means and standard errors of 16 flux measurements per forest age class are given for measurements made in January 2001, which is early in the wet season. These unpublished preliminary data are from an ongoing study by Davidson, Ishida, Alemeida, and Vieira.

**TABLE 4**
**Effects of Land Use Change in the Cerrado Region of Brazil**

| | $N_2O$ (ng N $cm^{-2}$ $h^{-1}$) | NO (ng N $cm^{-2}$ $h^{-1}$) | Reference |
|---|---|---|---|
| Native cerrado | Below detection | 0.5 | 19 |
| 5-year pasture | 0.3 – 0.9 | | 48 |
| 10-year pasture | 0.2 | | 49 |
| 20-year pasture | Below detection | Below detection | Verella et al. (unpublished data) |
| Soybean 100 days postplanting | 0.5 | | 49 |
| Corn after planting and fertilization | 1.5 | | 49 |
| Corn 100 days after fertilization | 0.2 | | 49 |
| Soybean in rotation with corn | 0.8 – 2.5 | | 48 |

soils[51,52,53], probably because the relatively dry Cerrado climate does not favor large $N_2O$ emissions. Irrigation is becoming more common, however, which could increase $N_2O$ emissions significantly[51].

## EFFECTS OF LAND USE CHANGE ON NITRIC OXIDE EMISSIONS

The effects of land use change on NO emissions (Table 5) is less clear than for $N_2O$. At Paragominas, Pará, NO fluxes from pastures and secondary forests were 20 to 45% those of the mature forest[10]. At Nova Vida, Rondônia, NO emissions were similar between forests and pastures during the wet season, but about ten times lower from pastures during the dry season[54]. On the other hand, a larger fraction of NO emitted from pasture soils is likely to escape to the atmosphere than occurs within forests, which have denser canopies that absorb more $NO_x$[14]. The pattern of NO emissions following forest clearing, burning, pasture establishment, pasture degradation, and secondary forest succession is likely to follow the same pattern described above for $N_2O$ emissions, except that NO is more important where there is a more pronounced dry season along the eastern and southern flanks of the Basin.

Preliminary results from a 20-year-old pasture near Brasília indicate that NO emissions are mostly below detection limits, with only sporadic detectable pulses (Verella et al., unpublished data; Table 4). In the absence of irrigation, we speculate that increases in N oxide emissions in row crop agriculture of this region might be more significant for NO emissions than for $N_2O$ emissions, due to the relatively dry climate. However, no measurements of soil NO emissions have been made, to our knowledge, in young pastures or in row crop agricultural fields in the Cerrado region.

**TABLE 5**
**Effects of Tropical Forest-Pasture Conversion on Soil Emissions of Nitric Oxide**

| | (kg NO-N $ha^{-1}$ $year^{-1}$) | | | | |
|---|---|---|---|---|---|
| | Mature Forest | Young Pasture | Old Pasture | Secondary Forest | Reference |
| La Selva, Costa Rica | 1 | 5 – 10 (2 – 10 years) | 0 – 1 (18 – 25 years) | 0.4 | 18, 24 |
| Pará, Brazil | 1.5 | 1.0 (0 – 13 years) | 0.6 (25 years) | 0.3 | 10 |
| Rondônia, Brazil | 1.4 | No data (1 – 3 years) | 0.2 – 0.6 (9 – 47 years) | No data | 54 |

## ACKNOWLEDGMENTS

Support for this effort was provided by LBA-Ecology project funded by the NASA Terrestrial Ecology Program, under Grant No. NCC5-332 to Eric Davidson and Grant No. IAG 5-10135-X to Richard Zepp, and EPA cooperative agreement, CAG-CR 827291-01-0, to Mercedes Bustamante.

| | | |
|---|---|---|
| Received: | July | 28, 2001 |
| Revised: | September | 27, 2001 |
| Accepted: | October | 3, 2001 |
| Published: | November | 21, 2001 |
